# Protocol for a randomized controlled trial of the Men in Mind training for mental health practitioners to enhance their clinical competencies for working with male clients

**DOI:** 10.1186/s40359-022-00875-9

**Published:** 2022-07-15

**Authors:** Zac E. Seidler, Michael J. Wilson, Nicholas W. Toogood, John L. Oliffe, David Kealy, John S. Ogrodniczuk, Jesse Owen, Andrew Mackinnon, Long Khanh-Dao Le, Cathrine Mihalopoulos, Jane Pirkis, Simon Rice

**Affiliations:** 1 Orygen, Melbourne, VIC Australia; 2 Centre for Youth Mental Health, The University of Melbourne, Melbourne, Australia; 3 Movember, Melbourne, Australia; 4 School of Nursing, University of British Columbia, Vancouver, Canada; 5 Department of Nursing, The University of Melbourne, Melbourne, Australia; 6 Department of Psychiatry, University of British Columbia, Vancouver, Canada; 7 Department of Counselling Psychology, University of Denver, Denver, USA; 8 Centre for Mental Health, Melbourne School of Population and Global Health, The University of Melbourne, Melbourne, Australia; 9 School of Public Health and Preventive Medicine, Monash University, Melbourne, Australia

**Keywords:** Gender, Training, Psychotherapy, Masculinity, Mental health, Trial, Men, Male, Practitioners

## Abstract

**Background:**

Although the proportion of men seeking professional mental health care has risen over the past two decades, on average, men continue to attend fewer sessions of psychotherapy and are more likely to drop out of treatment prematurely compared to women. Men account for three-quarters of suicide deaths; furthermore, over half of the males who die by suicide have engaged with mental health care in the 12 months prior to their death. These findings highlight a need to equip mental health practitioners with skills to improve male clients’ engagement and mental health outcomes. This article reports the protocol for a randomized controlled trial of *Men in Mind*, a self-paced online training program purpose-built to advance the clinical competencies of practitioners who provide psychotherapy to male clients.

**Methods:**

A randomized controlled trial with two parallel groups will be conducted. Participating practitioners will be randomly allocated, on a 1:1 basis, to the intervention group (*Men in Mind* training) or a waitlist control group. The primary outcome, efficacy of the training, will be evaluated by pre- to post-training (T1 to T2) changes in scores on the Engaging Men in Therapy Scale (EMITS) in the intervention group, relative to the control group.

**Discussion:**

This trial will provide evidence of the efficacy of *Men in Mind* training*,* as an interim step towards adjusting content and delivery of the intervention to maximize the potential for sustaining and scaling.

*Trial registration*: The trial was registered prospectively with the Australian New Zealand Clinical Trials Registry on 3rd December 2021 (ACTRN12621001669886).

**Supplementary Information:**

The online version contains supplementary material available at 10.1186/s40359-022-00875-9.

## Background

Although the proportion of Australian men seeking help for mental health problems has increased modestly in recent decades [1], the economic and social burden of men’s mental ill-health and suicidality in Australia remains significant, with men accounting for 75% of all suicide deaths [2]. Further, international evidence indicates that a large proportion of men who die by suicide seek help in the months prior to doing so [3, 4], cementing the need to critically reshape the nature of mental health services for help-seeking men.

A wealth of evidence documents men’s reluctance to seek help for mental ill-health, often due to structural and/or organizational barriers to care such as cost, length waitlists, and similar [5, 6]. Men’s hesitancy to approach services is also attributed, both in Australian and North American research, to traditional masculine socialization where rigid self-reliance and emotional restriction feature prominently [7–9]. Far less attention has been paid to those men who *do* seek help, overcoming a plethora of both structural and attitudinal barriers in the process. Recent work in Australia has highlighted that up to 40% of help-seeking men are self-motivated in their initiation of contact with mental health services [10]. Yet additional Australian evidence reveals room for improvement in the extent to which practitioners are providing a service that meets men’s needs [11]. A recent study of a sample of 1907 Australian men indicated premature dropout rates from therapy as high as 45%. The same study highlighted 27% of men accessed therapy once and did not return [12], potentially emblematic of the deterring effects of a dissatisfying therapy experience and potential for future reticence for help-seeking [13]. These patterns have also been observed in evidence from Spain [14] and Germany [15]. Reasons for treatment dropout among men reinforce the growing need to focus on the role of mental health practitioners in men’s mental health service engagement, as men most commonly reported dropping out due to a lack of connection with their practitioner [12]. Substantiating this, a growing body of work also explores challenges reported by practitioners in engaging and responding effectively to male clients [16–18]. In particular, a recent study focusing on practitioners’ experiences of challenges working with male clients highlighted engagement, helping men to communicate their affective experiences, and setting boundaries and handling in-session conflicts as common themes, especially for female practitioners [17]. In the absence of an available blueprint for the provision of male-oriented therapy, the onus will continue to fall on help-seeking men to adapt to a treatment framework which often fails to meet their needs, when equal attention should be paid to upskilling our practitioner workforce to better engage help-seeking men on their terms.

To date, efforts to upskill mental health practitioners in best-practice for reaching and retaining male clients in care have been limited to academic publication of recommendations sourced from experts in men’s mental health, both in North America and Australia [19–21]. In an effort to unify these disparate recommendations, a recent Delphi study [22] identified key competencies for practitioners working with men, including: the need to understand the intersection between masculine socialization and the experience of mental ill-health among men; and the need to implement strengths-based and goal-oriented therapeutic approaches within transparent and collaborative structures. These recommendations are largely encapsulated by the emerging construct of ‘gender competency’, which reflects practitioners’ capacity to understand and integrate male clients’ experiences of masculinity in relation to their presenting problem(s) and subsequent treatment needs [23, 24]. However, despite North American evidence that improving practitioners’ gender competency may lead to improved therapy outcomes for men [25], no such formal training program is currently available to mental health practitioners. Furthermore, to our knowledge, only two publications from the United Kingdom have reported the results of efforts to train health practitioners broadly in their engagement of male clients [26, 27]. Whilst these in-person training programs received positive feedback, to date a widely-accessible, scalable and mental health-specific training program for practitioners treating men does not exist.

To address this need, our team has developed an online training program, called *Men in Mind*, intended to improve mental health practitioners’ capacity to effectively engage and respond to male clients in psychotherapy. The five training program modules are structured around key domains derived from reviews of the literature [7, 20] and qualitative research with help-seeking men [11] and practitioners [17]. The development and theoretical background to the training program is described in depth elsewhere [28]. Pilot evaluation of the *Men in Mind* training, conducted in early 2021, found that the training program demonstrated acceptability, feasibility and potential efficacy in a sample of 196 practitioners [29]. Additionally, the pilot evaluation suggested training effects may differ by practitioner gender: a significant interaction between practitioner gender and pre-post intervention change in self-reported clinical competencies was observed. Female practitioners reported lower competency for engaging male clients relative to male practitioners in the pre-training survey, before improving to relatively equal levels with male practitioners post-intervention. No significant difference was seen for other demographic variables (e.g., years of experience), suggesting the unique influence of practitioner gender on self-reported competencies for working with men.

The next step in evaluation is to conduct a randomized controlled trial (RCT) to render evidence for the efficacy of the *Men in Mind* training in enhancing mental health practitioners’ self-reported clinical competencies in working with male clients. Here we report the protocol for a waitlist-controlled randomized trial of the *Men in Mind* training among a sample of Australian mental health practitioners.

### Aim

The objective of the trial is to determine the efficacy of the Men in Mind training on mental health practitioners’ self-reported clinical competencies for working with male clients.

## Methods

### Design

The study design is a randomized controlled superiority trial with two parallel groups. Participating practitioners will be randomly allocated, on a 1:1 basis, to the intervention group (*Men in Mind* training) or a waitlist control group. Randomization will be stratified by practitioner gender with three stratum: male, female, and a third stratum encompassing non-binary or self-identified genders. The trial will be sponsored by Orygen, Centre for Youth Mental Health, The University of Melbourne. Participant recruitment and consent, research assessments, and intervention will be conducted entirely online.

This trial will involve three occasions of measurement. The first will occur prior to randomization (pre-training/Time 1/T1); the second will occur at the conclusion of the 6-week training/waitlist period (post-training/Time 2/T2), this is the primary endpoint; and the third will occur at the conclusion of the 12-week follow-up period for the intervention group, and at the conclusion of the 6-week training period for the control group (follow-up/Time 3/T3).

### Participants

Eligible participants will be Australian-based mental health practitioners (i.e., psychologists, psychiatrists, counsellors, social workers, occupational therapists, mental health/psychiatric nurses, etc.), with experience treating male clients.

Participant inclusion criteria:

1. Mental health practitioner working in Australia;
2. Currently administering psychotherapy to male clients, either in person or via telehealth;
3. Fluent in English; AND
4. Provides informed consent to participate (see Additional file 1: Appendix A).

Participant exclusion criteria:

1. Undergraduate student.

### Materials

#### Demographic and clinical practice information

Participants’ demographic information will be collected at T1, in order to profile the participant sample and examine any subgroup effects post hoc. The following information will be collected: participants’ age; gender; profession; country of birth; identification as Aboriginal and/or Torres Strait Islander; primary theoretical orientation of practice (e.g. cognitive behavioral therapy; psychodynamic); length of time working as a practitioner; employment status (i.e., full or part-time); level of education completed; place (i.e., metropolitan or regional) and setting (e.g., private practice or community health) of work. Participants will also be asked whether they have completed any specific training program(s) related to working with men in therapy, and if so, will be asked to describe this in an open-text survey field. Participants will also be asked to indicate approximately how many individual clients they currently have on their caseload, alongside the proportion of these they estimate identify as male. These items will be incorporated as part of the cost analysis to understand the economic cost of the intervention per user.

### Primary outcome measure

Given the nascency of the field and the absence of published measures reporting mental health practitioner competencies related to working with male clients, self-reported clinical competencies will be measured using the Engaging Men in Therapy Scale (EMITS; [29]). The EMITS is a bespoke measure of practitioners’ self-reported capacity to engage and support male clients, which was developed for the purposes of the *Men in Mind* pilot study. In this pilot study the EMITS demonstrated satisfactory psychometric properties (*α* = 0.88–92 [29]). Example items include: *I know male-specific warning signs that might indicate suicidal action is imminent*; and *I am aware of specific strategies to use when a male client doesn't have the language to describe their emotions*. Participants respond on a five-point Likert scale questionnaire (1 = *strongly disagree*, 5 = *strongly agree*). The final score is the sum of responses to the 13 items (range: 13–65).

### Secondary outcome measures

The secondary outcomes in this trial encompass examining the effects of the *Men in Mind* training on practitioners’ self-reported confidence to engage male clients, alongside established measures of practitioners’ current clinical skills, professional self-doubt and self-efficacy, in addition to their experience of the training program. These measures are included to provide an indication of the effects of the *Men in Mind* training on practitioner outcomes more broadly than those assessed by the EMITS, which is directly tied to the content of the *Men in Mind* training.

### Confidence to engage male clients

Four additional items will be appended to the EMITS in order to specifically measure practitioner’s self-reported confidence in treating male clients experiencing issues specifically discussed in the training program (e.g., *I am confident in my ability to adapt my practice to help male clients identify and express their emotions*). These items will be rated on the same five-point Likert scale questionnaire (1 = *strongly disagree*, 5 = *strongly agree*). These four items will also be examined alongside the 13-item EMITS scale to analyse the reliability and validity of the scale as a 17-item measure.

#### Development of psychotherapists common core questionnaire (DPCCQ)

Effects of the training program on practitioners’ current clinical skills and professional self-doubt, will be measured using the DPCCQ [30]. This measure has been selected to provide an indication of the effects of the *Men in Mind* training on self-reported clinical competencies using a psychometrically-established scale. For this trial, the *current clinical skills* (7 items) and *professional self-doubt* (5 items) subscales will be used. Since their initial validation [30], these subscales have demonstrated good reliability (α = 0.87 and 0.77, respectively) across over 12,000 practitioners as part of an ongoing longitudinal study into practitioner development (D. Orlinsky, personal communication, July 7, 2021).

Participants will be asked to respond to each item on a 6-point Likert scale questionnaire (1 = *not at all/never*, 6 = *very much/very often*). Example items from the *current clinical skills* subscale include: *How effective are you at engaging clients in a working alliance*; *How effective are you in communicating your understanding and concern to your clients*; and example items from the *professional self-doubt* subscale including: *Lacking in confidence that you can have a beneficial effect on a client*; *Unsure how best to deal effectively with a client*; and *Demoralized by your inability to find ways to help a client*. All items will be subject to a minor adaptation (i.e., references to “client” will be replaced by “male client”) in order to orient participants to respond specifically according to their practice with male clients.

#### Counsellor self-estimate inventory (COSE)

Effects of the training program on counsellor self-efficacy will be measured using the *difficult client behaviors* subscale of the COSE [31]. This measure assesses practitioners’ perceptions of their self-efficacy in various therapy situations with 37 items across five subscales: *microskills* (12 items); *process* (10 items); *cultural competency* (4 items); *awareness of values* (4 items); and *difficult client behaviors* (7 items). The *difficult client behaviors* subscale is particularly relevant to the intervention under consideration in this study, given the items reflect practitioners’ self-efficacy in handling various situations discussed in the training program as common among male clients.

The COSE has demonstrated excellent reliability for both the total scale (α = 0.93–0.95) and the *difficult client behaviors* subscale (α = 0.80–0.85; [31, 32]). This subscale has also demonstrated convergent validity (with measures of self-concept) and divergent validity (with measures of state and trait anxiety). Participants will be asked to respond on a 6-point Likert scale questionnaire (1 = *strongly disagree*, 6 = *strongly agree*) according to the extent to which each item reflects their likely current performance in a given therapy situation. Example items include: *I am unsure as to how to deal with clients who appear noncommittal and indecisive*; *I may have difficulty dealing with clients who do not verbalize their thoughts during the counseling session*. Again, item references to “client” will be replaced by “male client” in order to elicit responses specific to participants’ practice with men.

### Additional measures

#### Objective audio-based competency assessment

##### Vignette activity overview

This study will also involve an objective audio-based competency test, to understand whether *Men in Mind* training participants display greater skills at responding to male clients relative to control group participants, comparing their pre- and post-training completion of the activity. Following a practice vignette to familiarize participants with the system, they will be presented with three 1–2-min video vignettes, each depicting one of the characters from *Men in Mind* demonstrating an issue discussed throughout the training program (e.g., restricted emotional expression). Participants will then be instructed to record their audio response to each video in turn, simulating their response as if they were in the therapy room with the client. The key aim will be to understand whether participants can incorporate discussion of masculinity alongside the skills taught in the *Men in Mind* training, and in doing so recognize where character statements in the vignette prompt some discussion of masculinity. An example of this is a vignette with Roger, a fictional male client who conveys a sense of disillusionment and defeat upon attending therapy, as he feels like his attendance is evidence that he is giving up. High-scoring responses will incorporate normalization of help-seeking, alongside validation of Roger’s concerns in light of the likelihood that masculine independence and self-reliance may have been the norm for him throughout his life. Once participants’ audio responses are submitted, data will be stored as part of the survey system for later download and coding.

##### Vignette response coding process

Participants’ vignette responses will be rated and coded according to the extent to which they reflect the skills and qualities taught in the *Men in Mind* training according to male-oriented practice. A broad coding framework has been developed in consultation with researchers conducting similar research focusing on multicultural competency [25, 33], given evidence that research paradigms aiming to train practitioners in multicultural competency can be readily adapted for training in gender competency.

Firstly, coders will rate whether or not the skills instructed in the prompts for each video, are actually demonstrated. In addition, responses will be coded according to the quality of the response, in terms of the extent to which each response is authentic, empowering and gender-oriented using Likert scale questionnaire measures. The rating scales to be used have been adapted from previous vignette-based work aiming to assess practitioner multicultural orientation (MCO; [25, 34]). Coders will also have the opportunity to enter any additional notes on the delivery of the response in an open-text field.

##### Response coding protocol

Response coding applying the above criteria will be undertaken by study Research Assistants; non-practitioners who will be trained, supervised, and blinded to the group allocation of participants. Coders will meet with study investigators and will be provided with a detailed explanation of the rationale for the study and their role as coders. Definitions of authenticity, empowerment, and gender-specificity will be provided as they relate to gender competency [25], in addition to providing coders full access to the *Men in Mind* training in order to familiarize themselves with the skills under study.

Training of coders will be covered via a half-day workshop, comprising detailed discussion of the rationale for the vignette activity. This will also cover the expected content and nature of responses indicating scores of the presence or absence of desired skills, in addition to scores indicating greater or lesser authenticity, empowerment and gender-orientation of responses. Following discussion of coding of example responses, the coding team will then be presented with test responses, and will be instructed to code each response independently. The team will then come together to discuss assigned codes. Any discrepancy in understanding of relevant codes will then be discussed as a team via an iterative education process with ongoing supervision.

To verify that coders are equipped to carry out this process, a subset of participant responses (5–10% of the total number, pending the amount of responses that are received) will be coded collaboratively with open discussion of correct codes and any discrepancies. Following this, another subset will be coded independently by coders, and investigators, who will then return and compare responses to ensure inter-rater reliability from the outset. Inter-rater reliability will be calculated statistically throughout the coding process using the intraclass correlation coefficient (ICC; ranging from 0–1), where values below 0.5 indicate poor reliability, between 0.5 and 0.75 moderate reliability, between 0.75 and 0.9 good reliability, and above 0.9 indicating excellent reliability [35]. Once coders achieve a minimum ICC of 0.75 they will be granted access to begin coding all responses.

#### Intervention experience measures

Participants' experience of the *Men in Mind* training will be measured using items from the E-Learning Satisfaction Scale (ELS; [36]). The 17-item ELS demonstrated acceptable reliability (overall α = 0.93), and evidence supporting content validity, criterion validity, discriminant and convergent validity, and nomological validity [36]. The *learner interface* (α = 0.90) and *content* (α = 0.89) subscales of the ELS will be used in this study.

Participants respond on a 7-point Likert scale questionnaire (1 = strongly disagree, 7 = strongly agree), with higher scores indicating greater learner satisfaction. Items will also include specific reference to the *Men in Mind* training, following the method applied by Harned and colleagues [37]. Example items include: *Men in Mind is easy to use*; *The content provided by Men in Mind is easy to understand*; *Men in Mind provides content that exactly fits your needs*; *Men in Mind provides useful content*.

Several training program experience items adapted from previous e-learning evaluation studies (e.g., Brownlow and colleagues [38]) will also be included to assess participants’ experience of the *Men in Mind* training. Participants will be asked to rate the following on the same scale as the ELS, from 1 (strongly disagree) to 7 (strongly agree): *I believe my current clinical practice will improve as a result of completing Men in Mind*; *I would recommend Men in Mind to other mental health professionals/colleagues*; *After completing Men in Mind, I feel more equipped to work with male clients in therapy*; *After completing Men in Mind, I am looking forward to working with more male clients*; and *After completing Men in Mind, I have been better able to retain male clients who have agreed to a course of therapy*. In addition, participants will be asked to respond to three free text entry items to gauge their experience of the training program. These items were applied in the pilot study and provided an opportunity for participants to provide more in-depth practical guidance for the iterative improvement of the content and learning experience in future: *In your own words, what was the best thing about the training program for you?* And, *In your own words, what do you think could be improved about the training program?* And, *In your own words, which population(s) of male clients do you feel more confident working with now, as a result of completing Men in Mind? (e.g., men experiencing suicidality, men experiencing difficulty with emotional communication, sexual minority men *etc*.)*

#### Goal assessment

We also aim to understand participants’ goals for implementing their learning from the *Men in Mind* training into practice, and the extent to which these goals are met over the course of the 12-week follow-up period. At T2, the intervention group will therefore be asked to respond to the following open-text item: *Now that you've completed Men in Mind, what are your three main goals for implementing the training into your practice?* Then, at T3, participants’ responses to this item will be presented back to them. They will then be asked to indicate whether they met their goals by responding with *No progress yet; Making progress* or *Achieved*, and to elaborate on why they did or did not meet them via an open-text survey item.

#### Qualitative evaluation

In addition, follow-up qualitative individual semi-structured interviews and focus groups will be completed with approximately 30 participants. These interviews will be transcribed and analyzed using qualitative methods such as thematic analysis [39]. This qualitative component will focus on understanding the acceptability, feasibility and potential efficacy of the training program, in addition to how and when learnings were implemented in practice, and suggestions for future refinement to aid in plans to scale the training program. Example questions will include: *How did you find the training program?; To what extent have you implemented the training in your professional practice?* and *What would you change about the training?*. We will seek to interview an even distribution of intervention and control group participants.

#### Cost analysis

In order to evaluate end-user expenses, as part of the cost analysis, several items will ask participants about their use of additional resources external to the training. For instance, participants will be asked whether, since completing *Men in Mind*, they have engaged with further resources relevant to the training, such as sourcing relevant books, academic articles, podcasts or other resources. If participants indicate ‘yes’, they will be asked to provide details about the specific resource and the approximate cost of access.

### Intervention

*Men in Mind* is an online training program for mental health practitioners working with men [28]. Development, creation, and hosting of the training program was funded by Movember, the world’s largest men’s health charity. The training program was designed for a wide variety of users (i.e., practitioners from mental health fields including psychology and psychiatry, counselling, social work, occupational therapy, mental health/psychiatric nursing, etc.). The goal of the training program is to increase practitioners’ competence and confidence in providing psychotherapy to male clients, by providing targeted training in the implementation of male-oriented adaptations to the process and delivery of psychotherapy. The content development and training program modules are structured around the following domains: (1) men’s gender socialization, masculinities, and gender as a determinant of mental health among men; (2) interactions between male client and practitioner gender socialization and specific considerations for male/female practitioners; (3) engagement and motivation strategies for male clients; (4) understanding and responding to men’s depression and (5) identifying and responding effectively to men’s suicidality. The training format is an interactive, skills-based process in which practitioners are encouraged to reflect on their own gendered experiences, their patterns of interaction with specific male clients, and the particular contexts in which they work. The content is contextualized through four male characters of different ages and backgrounds, who illustrate diverse examples of therapeutic situations over the course of the training program. In addition to text-based content, a suite of 41 professionally-produced, observational “skills-in-action” vignette videos populate the course, in addition to references to current academic and grey literature, practical “toolkit” resources and reflection exercises.

Development of the *Men in Mind* training involved extensive collaboration with learning and user experience design experts, alongside co-refinement of the final content of the intervention with various mental health practitioners and male consumers. These strategies likely contributed to the established acceptability and feasibility of the intervention, demonstrated in the pilot study where the *Men in Mind* training was completed in full by 158 practitioners from 207 initial consent responses (76.3% completion). The allotted 6 weeks for participants to complete the *Men in Mind* training was decided based on the pilot study, where 94% of participants completed the full training program by the conclusion of a 6-week period of access. As the training program is online and self-paced, during the trial, a programmed schedule of reminder emails will be sent to participants to remind them to engage with and complete the training program. These emails will be scheduled based on the stage of the intervention period, and will include personalized messages encouraging engagement with the training program and reminding participants of due dates. All reminder emails will be programmed and sent automatically by Strategic Data (our data management sub-contractor). Given the high rate of completion of the training program in the pilot study, augmenting the training program with the above strategy should be sufficient to enhance adherence.

All participants who are randomized to the intervention group will be given the same *Men in Mind* training, with no modification of the allocated intervention offered. Participants will be invited to complete the training program at their own pace over a 6-week period, and will be free to discontinue training and/or withdraw from the trial at any point. Note that, since no assessments take place during the intervention period, the intervention will only be discontinued at the participants’ volition. Participants will initially work through the modules of the *Men in Mind* training in the order in which they are presented, with no option to skip through the content; however, participants will be able to revisit previously completed content at their discretion until the conclusion of the 6-week intervention period. Post-intervention assessments will be sought from all participants who are randomized, regardless of whether they discontinue training. There are no restrictions on concomitant training in this trial.

### Comparator

Building on the *Men in Mind* single-group pilot study conducted in early 2021, a waitlist-control design was chosen for this trial. This will provide evidence of the efficacy of the *Men in Mind* training; in the pilot it was not possible to attribute observed effects to the intervention because there was no control group.

**Fig. 1:**
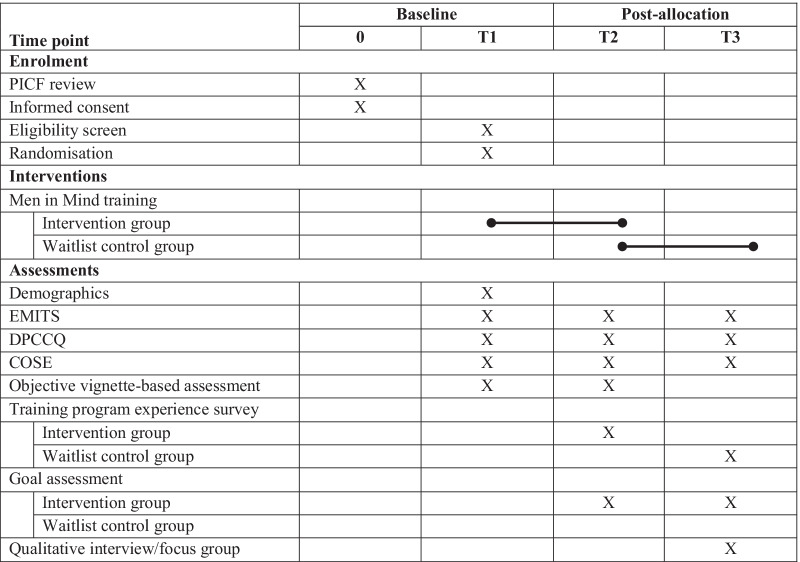
SPIRIT diagram for the men in mind trial

The trial will compare participants who receive the *Men in Mind* training with those who are waitlisted to receive the training program but who have not yet received it. A waitlist control condition was chosen for two primary reasons. First, all participants will be drawn from a register of practitioners who have previously expressed interest in undertaking the *Men in Mind* training. Therefore, this design will ensure that no participants are denied access to the intervention. An additional benefit, given this sampling, is that this design is likely to reduce dropout from participants who are not interested in completing an alternative, control intervention. Second, the waitlist-control design will capture current practice in the control group as closely as possible, in order to provide an estimate of the benefit of additional training. The SPIRIT diagram below summarizes the protocol for the trial (see Additional file 4: Appendix D for full SPIRIT checklist) (Fig. 1).

### Procedure

#### Recruitment

The recruitment target has been set at 380 participants. This number will allow detection of a small-to-medium size effect (Cohen’s *d* = 0.4) with 90% power and alpha 0.05, conservatively assuming a correlation between pre- and post-training scores of 0.5. This sample size allows for up to 30% attrition at T2 (primary endpoint).

Participants will be recruited from a register of practitioners who have voluntarily signed up to receive the training program at https://meninmind.movember.com. The target sample size of 380 participants is considered feasible, given that this register currently contains in excess of 2000 registrations, and the pilot study succeeded in recruiting 67% of those approached. Assuming this rate of consent holds in the trial, a minimum of 567 individuals will be invited to take part (allowing for a non-response rate of 33%). Recruitment will be facilitated by the dissemination of an initial study link via email to a selection of registrants. In addition, the link to the study may be disseminated via social media advertisements and distribution among the personal and professional networks of the investigators, where necessary.

### Consent

Informed consent will be provided online by participants, and required for enrollment in the trial. Participants will be provided with an online plain language statement as part of the initial assessment and asked to indicate their consent by responding to the following survey question: *If you agree with all of the information described here, please indicate your consent to take part by responding below*; presented with *Yes/No* response options. Participants who select *Yes* will be automatically directed to the initial survey assessment; those that select *No* will be directed to close the survey. Participants will be invited to contact the Principal Investigator to discuss any issues or questions that arise during the consenting process.

### Allocation

Participants will be randomized in a 1:1 ratio to either the intervention group (*Men in Mind* training) or control group (6-week waitlist). Permuted block randomization will be performed, using variable sized blocks, with stratification by participant gender (three strata: male, female and non-binary/self-identified gender). The randomization schedule will be generated before recruitment by an investigator unconnected with the day-to-day running of the trial.

Randomization will be programmed by our data management subcontractor, Strategic Data. The allocation schedule will be created by the study statistician and transferred directly to Strategic Data, unseen by the researchers. The eligibility of any individual participant will be automatically assessed and determined prior to group allocation, such that allocation cannot influence the decision to include or exclude the participant.

Online questionnaire data collection will be undertaken by Strategic Data who will use a purpose-built online platform for the survey. They will generate unique URLs for each participant based on their email address, so that participant responses can be connected across all surveys. At the end of trial, Strategic Data will provide the questionnaire data to the researchers in de-identified, blinded format.

### Blinding

Participants will be aware of their group allocation. The research team members, including data analysts, will be blinded to the group allocation of individual participants. The only exception to this is that the Principal Investigator will be unblinded in order to facilitate communication with participants where necessary over the course of the study (e.g., if participants raise questions over the course of the trial regarding technical difficulties etc. this will naturally expose the group to which participants are allocated). All participant data in this study will be self-reported, and the final data set will be completely de-identified, so it is unlikely that any participant contact during the trial will be able to be used to identify participants in the final data set. Observer-rated measures and data analysis will be conducted by blinded members of the research team.

If a participant withdraws from the trial, the Principal Investigator (unblinded) will ensure that participant is not contacted for the next assessment. Participants can contact the researchers via the Principal Investigator’s e-mail address, which is provided on the Plain Language Statement. The Principal Investigator will also seek to determine the reason for the participant’s withdrawal in order to manage the duty of care to participants and troubleshoot any concerns where possible.

### Data collection

**Fig. 2:**
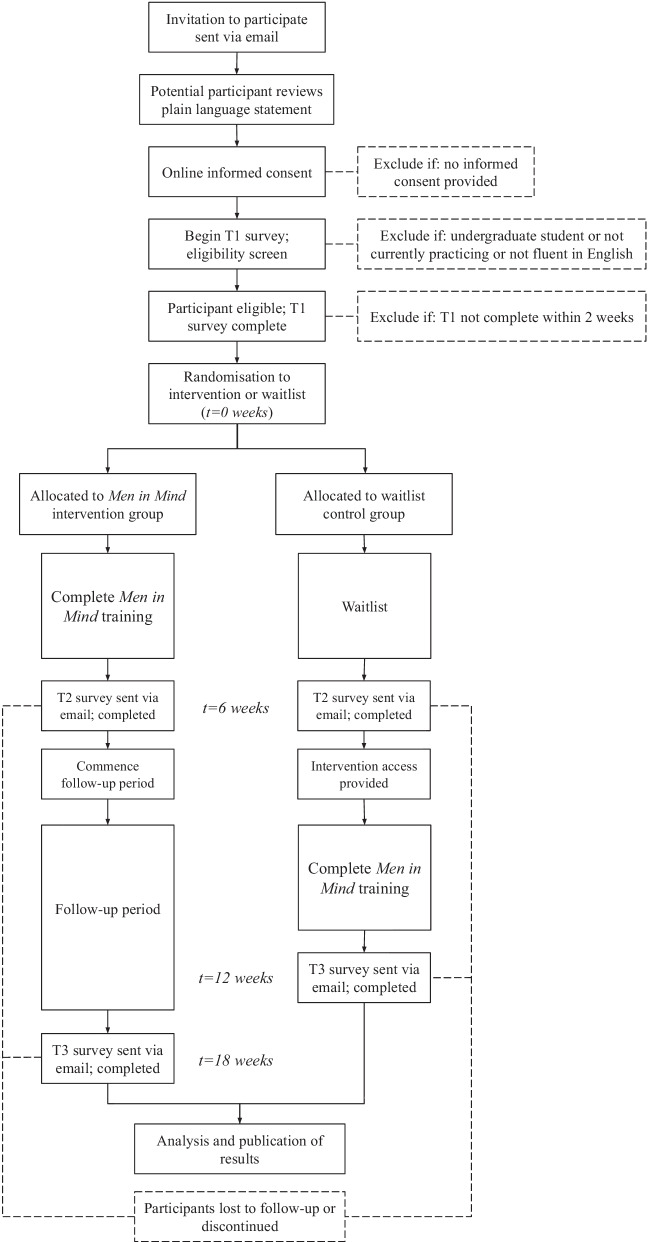
Men in mind trial participant flow diagram

The flow diagram above summarizes the data collection procedures in the trial. Initial invitations to take part in the trial will be sent via email. The invitations will contain a brief overview of the study and a link to the plain language statement and consent form as part of the pre-training (T1) survey. Participants will be given a 2-week period (with reminders) within which to provide consent to participate before they are considered a non-responder (Fig. 2).

Participants who click through the initial link will be presented with a study plain language statement and consent form. After reading the plain language statement, participants will be asked to indicate their consent by responding to a yes/no survey item. Those who do not consent will be thanked for their time and directed out of the survey. At the end of the 2-week period, should the recruitment target not be met, an additional randomly-selected cohort of individuals from the available register of practitioners who previously expressed interest in completing the *Men in Mind* training will be invited to take part, and also allowed 2 weeks to respond. Consenting participants will then be presented with the full T1 assessment. This will contain self-report measures detailed above, in addition to the objective vignette-based competency assessment. Upon completion of the pre-training assessment, participants will be randomized to either the intervention group or the waitlist control group. Gender entered as part of the T1 survey demographic items will be used as a stratification factor (i.e., male, female and a third stratum encompassing non-binary or self-identified genders) in the randomization process given the significant gender differences in outcomes from our pilot findings.

The intervention group (referred to as “Group A” in participant-facing documentation) will be provided immediate access to the training program for a period of 6 weeks after they complete the T1 assessment. During this time, regular automatic email reminders will be sent to prompt module completion regardless of progress, until participants complete the training program. At the end of the 6-week training period participants will be sent the post-training assessment link (T2) via email. Participants will receive reminders over a 2-week period until the post-training survey is completed. At the conclusion of the 6-week training period, the intervention group will also be informed of the commencement of their follow-up period of 3 months (12 weeks total), and instructed to expect to receive a third and final survey after this period of time. A brief ‘check-in’ style contact will be sent to participants half-way through this follow-up period, as a strategy to minimize attrition and ensure participants’ correct contact details are on-hand. Once the T3 survey is sent, participants will have a 2-week period to complete this with reminders sent until the T3 survey is submitted. Participants who do not complete the T2 survey will still be invited to complete the T3 survey.

After submitting the pre-training survey, the control group (“Group B”) will be informed that their access to the training program will commence after a 6-week period, and following submission of a second survey (T2). During this period, a brief ‘check-in’ style contact will be sent to maintain engagement, equalize contact and, hopefully, minimize attrition and ensure participants’ correct contact details are on hand for all subsequent assessments. Once the T2 survey is sent, participants will receive reminders to complete this over a 2-week period. Access to the training program will be provided automatically on submission of the T2 survey. The control group will be provided access to the training program for a period of 6 weeks, after which their access will be closed off. At the conclusion of their 6 weeks of access a final T3 survey will be sent via email to the control group.

### Data management

All data will be stored indefinitely on secure, password-protected servers in Australia. A unique study identifier will be generated by Strategic Data for each participant and will link responses across time-points. In the first instance, data will only be accessible to members of the research team. However, at the discretion of the Primary Sponsor and Principal Investigator, de-identified data may be shared with researchers who present a methodologically sound proposal and have ethics approval to conduct research using de-identified data obtained in this study.

In addition, the research team will undertake data monitoring over the course of the trial in collaboration with the data collection sub-contractor, Strategic Data, with consultation from the trial statistician. Responses will be inspected to determine data integrity.

### Retention

The strategies designed to enhance adherence to the intervention will also serve to promote participant retention and complete follow-up. The programmed schedule of reminder emails will encourage intervention group participants to continue engaging with the training program. Control group participants will also receive emails, which will remind them of the ‘countdown’ to their intervention access and are designed to keep them engaged throughout the waitlist period. These strategies will likely reduce the frequency of losing participants to follow-up after randomization. Following the protocol applied in the pilot study, once T2/T3 assessments have been sent to participants, they will receive emails at regular intervals encouraging them to complete these assessments. Furthermore, control group participants will only be provided access to the training after they have completed their T2 assessment; this incentive should reduce attrition in this group and maximize the available data for the primary outcome.

### Statistical analysis

Analyses will be undertaken on an intention-to-treat basis and will include all participants in the group to which they were randomized (regardless of actual receipt or uptake of the intervention or withdrawal from the study). Mixed-model repeated measures analyses will be used because of the ability of this approach to include participants with missing data. The model will include factors of study condition (intervention or control group), occasion of measurement (T1, T2 and T3), and their interaction. Analyses will include the effect of the stratification variable, gender, with associated model parameters being retained if they are statistically significant.

### Primary outcome

The primary outcome will be assessed by a planned comparison of the difference between groups in change of the primary outcome variable (EMITS) from T1 to T2. This test will be undertaken with an alpha of 0.05. An unstructured residual variance–covariance matrix will accommodate within-participant dependency. Tests of significance will use appropriate degrees of freedom adjustment where necessary (e.g., the Kenward-Roger method based on the observed information matrix). Where necessary, transformation of the outcome variable will be undertaken to ensure distributional assumptions of the model are met.

### Secondary outcomes

Analyses of secondary outcome variables (Confidence items, DPCCQ and COSE subscales) will follow the same methods as the primary outcome. These analyses will be subject to appropriate adjustment for multiple testing. Secondary outcomes will also include change in the primary and other outcome variables from baseline (T1) to follow-up (T3) to inform the outcome pertaining to retention of learning. (Note that change in the intervention group over this period cannot be compared to the waitlist as the latter group will be provided with access to the intervention after T2.) The magnitude of change within both active and control groups from T2 to T3 will be also estimated to reflect retention of learning. T3 outcomes in the waitlist group will be compared to their T2 scores in order to estimate change in this group after they have accessed the training program. The extent of change will be contrasted to T1–T2 change attributable to the training program in the intervention group. Subject to qualifications arising due to attrition and natural drift over time, these analyses will stand as a quasi-replication of the primary outcome of the trial.

### Exploratory analyses

#### Examining the EMITS as a 17-item scale

The four items assessing confidence to engage male clients will be examined alongside the 13-item EMITS scale, to ascertain the reliability and validity of the scale as a 17-item measure to inform usage in future studies.

#### Subgroup analyses

Subgroup analyses may be conducted for variables including (but not limited to) participants’ profession, education level, and years of experience. These analyses will be reported as exploratory analyses separate to the primary outcome.

#### Analysis of objective audio-based competency assessment data

Once vignette responses are coded according to the presence/absence of desired skills, and quality of responses, the T1-T2 change in these variables will be compared between the intervention and control groups to examine the efficacy of the *Men in Mind* training at improving practitioners’ competencies more objectively.

#### Intervention experience analysis

Descriptive statistics and inductive thematic analysis will be used to analyze responses to the closed and open-ended questions encompassing participants’ experiences of the *Men in Mind* training.

#### Cost analysis

A cost analysis of the intervention will also be conducted. This will draw on the training-program development and delivery costs, including staffing, technology and course material, alongside costs associated with evaluating the intervention, to understand the holistic cost of delivering the *Men in Mind* training per user.

### Monitoring

This trial has received ethical approval from the University of Melbourne’s Faculty of Medicine, Dentistry and Health Sciences Human Research Ethics Committee (ID: 22,618). The trial has also been prospectively registered with the Australian New Zealand Clinical Trials Registry (ID: ACTRN12621001669886).

The Principal Investigator will oversee and coordinate all aspects of the trial and consult the other investigators associated with the trial at key decision points. There is no formal data monitoring committee for this trial. However, whilst no auditing is pre-planned, the study may be subject to routine auditing by the Sponsor (Orygen) and/or approving ethics committee (The University of Melbourne Psychology, Health and Applied Sciences Human Ethics Sub-Committee). In addition, the research team will undertake data monitoring over the course of the trial in collaboration with the data collection sub-contractor, Strategic Data, with consultation from the trial statistician. Responses will be inspected to determine data integrity. No interim analysis will be conducted.

The potential risks of participating in this trial are expected to be limited to inconvenience due to the time commitment involved. Nonetheless, a potential risk relates to participants experiencing distress as a result of the nature of the content of the course. This relates to the latter modules of the course as these are dedicated to discussion of men’s experiences of depression and suicidality. Given the prevalence of male suicide, the content of these modules may be distressing to participants. However, several factors may mitigate this potential risk. First, participants will be alerted to the presence of this content in the plain language statement, and will therefore be unlikely to be surprised by the content of the course as they progress through it. Second, the content of Module 5 will also be clearly sign-posted in the course, and this content will be presented alongside relevant helplines and mental health support services for participants to contact in the event that distress has arisen. Any participants who do disclose distress related to their participation in the trial will be contacted by the Principal Investigator, who will discuss withdrawal from the trial and recommend appropriate services, if necessary.

### Dissemination plan

All participating practitioners will be provided with a summary of the findings of the trial at its conclusion. Findings will also be reported in journal articles and presented at scientific conferences, alongside internal and external reporting and public forums where requested. Once the trial is complete, Movember will aim to iterate and scale the training program into their international markets (e.g. Canada, UK, USA), while making it more broadly available for all mental health practitioners in Australia.

## Discussion

In light of growing evidence of unique challenges experienced by mental health practitioners in engaging their male clients, men’s mental health scholars continue to provide broad recommendations for adaptations to treatment in order to facilitate engaging, tailored care for men. The translation of this knowledge into the *Men in Mind* training program represents an important step to advance the field, as the first mental health practitioner-specific, scalable intervention, focused on enhancing practitioner competencies for working with men. Following promising results from the *Men in Mind* pilot study, it remains to be confirmed whether the program confers tangible benefits to practitioners’ self-reported clinical competencies for working with male clients. This RCT is therefore well-placed to provide further insight regarding the efficacy of the *Men in Mind* training.

### Trial methodological considerations

Questions concerning the feasibility of delivering this trial per-protocol largely stem from the potential that recruitment targets may not be met, and attrition may limit confidence in findings if analyses need to account for large proportions of missing data. The accessible, self-paced and online delivery of the training program is anticipated to conform to the ways in which mental health practitioners prefer to engage with continuing professional development activities [40], hopefully mitigating these risks. In addition, engagement with online learning among practitioner workforces has likely been further normed given the context of the COVID-19 pandemic, justifying the delivery of the *Men in Mind* training online to facilitate wide access by practitioners. As described above, the participant sample will be drawn from a pool of practitioners who previously registered interest in receiving the *Men in Mind* training. Concerning attrition across the three occasions of assessment, whilst inevitable, we are confident on the basis of the minimal attrition observed in the *Men in Mind* pilot study that this outcome will not severely impact the primary outcome measurement across T1-T2 assessments. Attrition from T2-T3 particularly in the intervention group may be greater due to participant fatigue, alongside the longer period between assessments. However, the choice of a 12-week follow-up period aligns with the final follow-up periods reported in previous online practitioner training evaluation studies, where observed post-training to follow-up attrition is low (e.g., 0% attrition in Dimeff et al. [41]; 6% attrition in Ehrenreich-May et al. [42]; 2% attrition in Harned et al. [37]). All this combined engenders confidence in the likelihood of recruitment targets being met in this trial.

Another potential limitation concerns the extent to which the findings from this trial can be translated to practitioner populations more broadly. As described, all participants will have previously expressed interest in receiving the *Men in Mind* training, resulting in a potentially ‘warm’ sample whose outcomes may differ from those observed among practitioners with less eager interest in adapting their practice according to their male clients’ gender socialization. Yet, given the plan for the *Men in Mind* training to be situated alongside other continuing professional development opportunities for practitioners, it is anticipated that in future those who choose to engage with the *Men in Mind* training will have some interest in men’s mental health. As such the findings from this trial will, at least in this regard, be indicative of expected outcomes among practitioners in the general Australian population when the *Men in Mind* training is available more widely. Moreover, whilst we will be able to tally numbers of practitioners who refuse to participate, due to the constraints of the trial methodology, it will not be possible to ascertain the characteristics of those practitioners who choose not to take part in the study. We will also not be able to control potential contamination between groups, such as situations where multiple practitioners from the same service are randomized to different groups.

In terms of strengths of the study, the inclusion of the vignette-based activity also represents a novel attempt to understand the impact of the training program more objectively, through the coding of participants’ audio responses. Whilst vignette-based assessments have been used in previous training evaluation studies, this methodology rarely extends to the objective evaluation of *adaptations* to practice according to client characteristics, as is the focus of the *Men in Mind* training. Available studies have applied vignettes to measure practitioner proficiency in delivering a novel manualized therapy following, for example, training in dialectical behavior therapy [41], cognitive behavior therapy for adolescent panic disorder [42] and exposure therapy for anxiety disorders [37]. The use of vignette-based assessment to understand the impact of the *Men in Mind* training on practitioners' style and delivery of therapeutic responses with fictitious male clients therefore represents a novel step forward for the field.

### Future directions and conclusions

Findings from this trial will inform the future refinement and potential dissemination of the *Men in Mind* training. In particular, examining any subgroup differences in change on outcome measures following exposure to the intervention, alongside qualitative insights gleaned from interviews and/or focus groups with participants, will shed light on potential avenues to specifically target the scaled rollout of the *Men in Mind* training among populations of practitioners who may benefit most. For example, practitioners working in youth-specific mental health services may find areas of the training program that are particularly beneficial, especially in light of high service discontinuation rates among young men [12, 43]. In addition, the *Men in Mind* pilot evaluation revealed an interaction between change on the EMITS and practitioner gender, where female participants began with significantly lower self-reported skill with male clients relative to male practitioners. Whilst the trial will incorporate stratification based on practitioner gender to account for these effects, the in-built qualitative evaluation will shed light on further ways in which female practitioners might benefit from the training program.

To conclude, there undoubtedly exist a number of structural and organizational barriers to help-seeking that are known to affect men’s uptake of mental health services [5, 6]. Yet establishing the efficacy of the Men in Mind training will aid the field by illuminating practitioner training as a viable avenue to ensuring the increasing numbers of help-seeking men are met with services that meet their needs.

## Supplementary Information

**Additional file 1:** Participant consent form.

**Additional file 2:** Trial ethical approval.

**Additional file 3:** Funding statement.

**Additional file 4:** SPIRIT checklist.

## Data Availability

All participant data will be stored electronically by Strategic Data, in accordance with strict, contractually agreed security measures. Upon the completion of data collection, Strategic Data will supply the deidentified data to the named researchers. Anonymized, participant-level data may be made available upon request to external researchers, with prior ethics approval, on the basis of a clear research methodology employing the data. Any such data sharing will remain at the discretion of the Sponsor and Principal Investigator. The study protocol will be available at the Australian New Zealand Clinical Trials Registry (ANZCTR). Any amendments to the protocol will be provided to the ANZCTR and will also be communicated to all trial researchers.

## Abbreviations

EMITS: Engaging men in therapy scale
DPCCQ: Development of psychotherapists common core questionnaire
COSE: Counsellor self-estimate inventory
ICC: Intraclass correlation coefficient
ELS: E-learning satisfaction scale
