# Supplementary material for: Protocol for a randomized controlled trial of the Men in Mind training for mental health practitioners to enhance their clinical competencies for working with male clients
Source: BMC Psychol. 2022 Jul 15;10:174. doi: 10.1186/s40359-022-00875-9 (PMC9288021; doi:10.1186/s40359-022-00875-9)
Supplement: Supplementary file 1 — Additional file 1: Participant consent form. [file 40359_2022_875_MOESM1_ESM.docx]

**Information Statement – Men in Mind training**

Centre for Youth Mental Health

Faculty of Medicine, Dentistry and Health Sciences

The University of Melbourne

## ***Project Title:*** *Men in Mind: Randomised controlled trial of an online mental health clinician training program for engaging men in therapy*

## **Responsible Researcher:** Dr Zac Seidler; [zac.seidler@movember.com](mailto:zac.seidler@movember.com)

### Introduction

Thank you for your interest in participating in this research project. Please take the time to read this information carefully and seek clarification about anything you don’t understand or want to know more about.

In order to participate, you must be a mental health practitioner currently providing psychotherapy to male clients in Australia. Fluency in English is also required in order to take part.

Your participation is voluntary – you don’t have to participate. If you begin participating, you can also stop at any time. The contact details of the research team will be provided should you wish to withdraw later on.

This project has been approved by the The University of Melbourne, Medicine, Dentistry and Health Sciences Human Ethics Sub-Committee (22618). This project is funded by Movember (development of the training) and the Australian Government’s Million Minds Mental Health Research Mission (evaluation of the training).

### What is this project about?

This project represents a partnership between Movember and Orygen. Movember is the world’s leading men’s health charity, and Orygen is a world-leading youth mental health clinical service and research organisation at the University of Melbourne.

Recognising that many male clients have complex needs and require gender-sensitive engagement in psychotherapy, and often practitioners are unaware of ‘best practice’ when it comes to engaging male clients, Movember have funded a partnership with Orygen to develop a world-first online training program, called *Men in Mind*. The 5-module online training course aims to upskill therapists in engaging male clients in therapy, implementing male-oriented adaptations to treatment, and responding more effectively to depression and suicide in men. With this study, we are hoping to investigate the effectiveness of the training at improving therapists’ capacity to engage and connect with their male clients.

Please note that the training program does not cover information specifically related to engaging and responding to male clients identifying as transgender in mental health treatment.

### What will I be asked to do?

If you agree to take part, once you complete an initial survey for us, you will be allocated to complete the training straight away (i.e., group A), or allocated to receive the training after a period of 6 weeks (i.e., group B). Which group you are allocated to is determined at random (like flipping a coin), but if you agree to take part, **you will definitely receive the training.**

To investigate the benefits of *Men in Mind*, we will ask all participants to complete three short surveys at various stages of the study. Surveys will all be accessible via the same link you used to access this form, and we will send reminders in case you lose the link. The first survey will come immediately after you submit this consent form. Once you complete the first survey, you will be randomly allocated to either group A or group B.

**If you are in group A**, you will be given access to the training straight away, and will be asked to complete two surveys after the course (a post-training survey 6 weeks after you start the training, and a follow-up survey 3 months after you finish the course).

**If you are in group B**, you will be provided access to the training once you complete a second survey, which will be sent 6 weeks after you complete the first survey. You will then be asked to complete a final survey, which will be sent 6 weeks after you start the course.

The surveys will involve demographic information, such as your age, gender, profession and similar (first survey only). In addition, all three surveys will include questions related to your experiences working with men, skills related to engaging and responding to male clients in therapy, and some questions about your current clinical skills and confidence in relation to working with male clients.

The first and second surveys will also include a scenario-based activity. This will involve you watching a series of 3 videos of male clients in various therapy situations. You will then be asked to record an audio-only response to each client, as though you were in therapy with them. This is not intended to judge your quality as a therapist, but is more for us to understand how well we are teaching the content of Men in Mind.

The training is hosted on an external learning system, **involves 5 modules and takes a maximum of 8 hours, and you will have 6 weeks in which to complete the training, but you might finish it sooner than this.** Completing the training will involve reading content and watching videos of various skills in action, in addition to completing reflective journaling activities. Please note that you should not enter any sensitive or identifying information about clients you’ve worked with in these journaling activities.

**The total time commitment involved is expected to be around 9 hours (~8 hours completing the online training; and an additional maximum of an hour completing the research assessments).**

The diagram below summarises everything involved in this study.


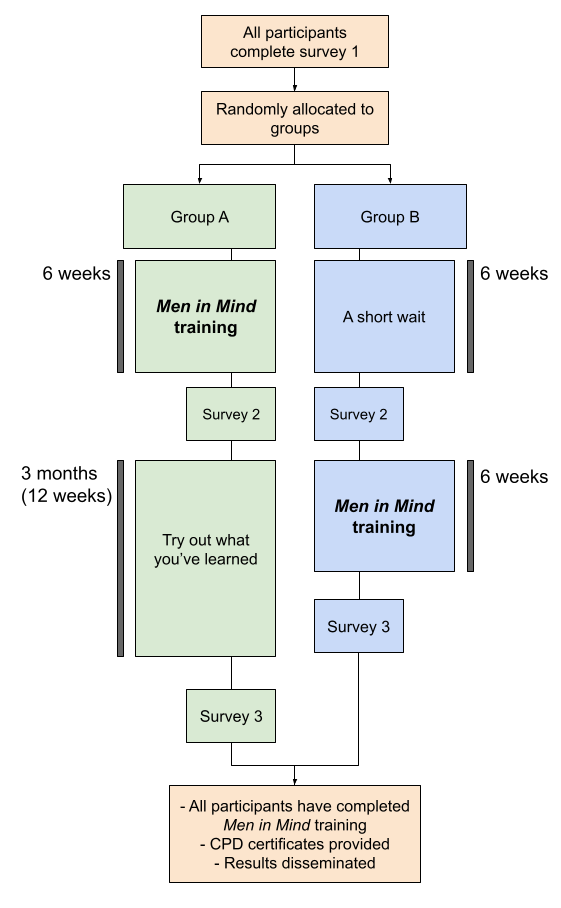


### When will I be contacted?

We will contact you with email reminders to continue working through the training and/or to complete the surveys over the course of the study. We will also contact you via email with updates about the study including results, and opportunities for future research participation.

A research study team member or a representative of the Orygen communications team may contact you asking for your thoughts on the research project or your lived experience. You are free to say no to their requests, or to provide comments anonymously. Your comments may be used to promote the research study’s findings on social media, in a news article or other communications materials. If you do not wish to be contacted for communications purposes, please let a member of the study team know.

### What are the possible benefits?

The *Men in Mind* training is a world-first initiative designed to improve clinician’s competency in counselling male clients. Your participation helps us understand the effectiveness, such that it can be scaled up and rolled out to therapists around the world. Aside from receiving the training program as free CPD, and any learning or skill development you experience, no direct benefits of participation are anticipated for you.

### What are the possible risks?

The main risk for you is inconvenience due to the time commitment involved in the study (approximately 9 hours in total).

An additional risk involves the sensitive nature of the content of the course, particularly in Modules 4 and 5, which focus on men’s depression and suicidality. The content of these modules may be distressing. We encourage you to ensure you have adequate self-care strategies in place prior to and following completion of these modules. The research team contact details, and some support services, are also be provided should you require direction to further support.

### Will I be reimbursed?

We encourage you to claim the time spent completing the training program in this study as part of your Continuing Professional Development (CPD) hours. We will also send out a certificate to all participants who complete the training via email. No other reimbursement will be offered.

### How confidential is the information I provide?

All information you provide when completing surveys will be stored securely on password-protected Australian servers by Strategic Data, an external data management organisation. Strategic Data will have access to your name and email address in order to facilitate study reminders; however this information will never be stored together with your study data, so your responses won’t be identifiable. All data you provide will be stored by Strategic Data against a study identifier (ID) that is unique to you, so will be completely confidential. You will be provided with a unique link via email to access all tasks involved in this study, and each participant will have their own link.

At the conclusion of the study, all data collected by Strategic Data will be securely transferred to the research team at Orygen and stored indefinitely on password-protected servers, to which only the research team will have access.

In addition to this, while you are completing the training, some data will be collected regarding time spent on given pages of the training, interaction with training components, and similar. This will be stored securely as part of a Google Analytics plugin to the training software. This information will eventually be downloaded to secure, password-protected Orygen servers for analysis.

### What will happen to information about me?

Personal information that you give us as part of your participation in the project won’t be passed on to anyone outside the research team without your permission, subject to any legal reporting requirements. Also, any research data may be subject to monitoring or auditing to ensure procedures are in compliance with regulations. This may be undertaken by the approving ethics committee, or the sponsor of the study (Orygen).

At the conclusion of the study, your anonymous data will be analysed along with other participants, and likely published in a scientific journal alongside internal and external reports. In any publication, information or data will be provided in such a way that you cannot be identified (i.e., no names or contact information will ever be published). Anonymous aggregate data may be provided to Movember for reporting purposes and future training development. Your anonymous research information will be kept indefinitely for these purposes.

Researchers from other universities or organisations may request to use the data from this study for their own research. If we agreed to pass on the data, the data we would provide would never include your personal details, such as your name and contact details. The data we would provide would only be the responses to the questionnaires (your responses to the vignette activity and qualitative interview/focus group data would never be passed on). There would be no way to identify you through this data. We would approve or reject researchers’ written requests to use this data on a case-by-case basis. Only research that has gained ethics approval from a human research ethics committee, as needed, would be considered.

### Can I access information about me?

When analysing study data, we will be compiling all participants together, so it won’t be possible to provide individual results to any participant.

### How will I be informed of the final results of this research project?

Once the project has been completed, a summary of findings will be disseminated to participants via email.

**Who can I contact if I have any concerns about the project?**

### This project has human research ethics approval from The University of Melbourne (project ID 22618). If you have any concerns or complaints about the conduct of this research project, which you do not wish to discuss with the research team, you should contact the Research Integrity Administrator, Office of Research Ethics and Integrity, University of Melbourne, VIC 3010. Tel: +61 8344 1814 or Email: [research-integrity@unimelb.edu.au](mailto:research-integrity@unimelb.edu.au). All complaints will be treated confidentially. In any correspondence please provide the name of the research team and/or the name or ethics ID number of the research project.

**What if I need support?**

If you need support for your own mental health, you can contact one of the services below:

Lifeline: 13 11 14

Beyond Blue: 1300 22 4636

If you need assistance with direction to appropriate support services, please email [zac.seidler@movember.com](mailto:zac.seidler@movember.com).

**Summary**

If you agree to take part in this study, you are agreeing to the following:

| **Yes (please mark X)** | **Item** |
| --- | --- |
|  | Complete three surveys in total at varying time points across the study including scenario-based activities (see diagram above). |
|  | Once provided access, complete *Men in Mind* in your own time over a period of 6 weeks. |
|  | My anonymous study data will be kept for research purposes. I won’t be identifiable as my contact details will be stored separately from study data. |

If you agree with all of the information described here, please indicate your consent to take part in the study by responding below:

| **☐ YES, I consent to take part in this study.** |
| --- |
| **☐ NO, I do not consent to take part in this study.** |
