# Supplementary material for: Protocol for a randomized controlled trial of the Men in Mind training for mental health practitioners to enhance their clinical competencies for working with male clients
Source: BMC Psychol. 2022 Jul 15;10:174. doi: 10.1186/s40359-022-00875-9 (PMC9288021; doi:10.1186/s40359-022-00875-9)
Supplement: Supplementary file 2 — Additional file 2: Trial ethical approval. [file 40359_2022_875_MOESM2_ESM.pdf]

Office of Research Ethics and Integrity

THE UNIVERSITY OF MELBOURNE

Human Ethics Application Approval

ATTENTION: DR Zac Seidler

3277 - Centre for Youth Mental Health
140 - Medicine, Dentistry and Health Sciences
The University of Melbourne

Research Application

**Reference Number:**	2021-22618-23112-3
**Project Title:**	Men in Mind: Randomised controlled trial of an online mental health clinician training program for engaging men in therapy

Dear DR Zac Seidler,

Thank you for your response to queries raised by STEMM 3.

The Committee agreed to **approve** the application on the basis that it meets the requirements of the National Statement on Ethical Conduct in Human Research (2007, Updated 2018). Please see overleaf, *Summary Details for the Approved Human Ethics Project* and *Conditions of Approval*. It is your responsibility to ensure that all people associated with the Project are made aware of what has been approved.

Desk-based elements of your project and face-to-face research can commence now, as can data collection that can be conducted online or via telephone, subject to necessary approvals or amendments to ethics applications.

Please consult the COVID-19 website for research guidance, FAQ and updates. https://staff.unimelb.edu.au/covid-19-response/research-activity

If you have any queries on these matters, or require additional information, please contact me using the details below. Please quote the ethics ID number and the title of the Project in any future correspondence.

Yours sincerely,

MS Belinda Kelly

Research Ethics Officer

Human Ethics Team
Office of Research Ethics and Integrity | Research, Innovation & Commercialisation
Level 5, Alan Gilbert Building, 161 Barry Street, Carlton
The University of Melbourne, Victoria 3010, Australia
T: (03) 9035 9095 E: belinda.kelly@unimelb.edu.au

Summary Details for the Approved Human Ethics Project

Project Title:	Men in Mind: Randomised controlled trial of an online mental health clinician training program for engaging men in therapy
Reference Number:	2021-22618-23112-3
Approval Date:	29/10/2021
Expiry Date:	29/10/2024
Responsible Human Ethics Committee	STEMM 3
Project Supervisor	DR Zac Seidler
Other Investigators	A/PROF Simon Rice, MR Michael Wilson, MR Nicholas Toogood, PROF Jane Pirkis, PROF Andrew MacKinnon
External Investigators	Prof Cathy Mihalopoulos, Prof John Oliffe, Prof John Ogrodniczuk, Prof Jesse Owen, Dr David Kealy, Dr Long Le, Glenn Lee

Documents table:

Document Type	File Name	Date	Version
Other	F21-407 Reviewer Comments & Response (1 of 2)		
Other	F21-407 RRC Memo and Response (2 of 2)		
Full protocol (for medical research)	1. Men in Mind trial - study protocol	20/09/2021	1
Consent form	2. Men in Mind trial - standard PICF	20/09/2021	1
Consent form	3. Men in Mind trial - interview focus group PICF	20/09/2021	1
Questionnaire(s) and/or survey instrument(s)	4. Men in Mind trial appendix - all items	20/09/2021	1.0
Recruitment materials	6. SEP_Men In Mind_Version1_170821_ethics version	20/09/2021	1.0
Other	5. Men in Mind trial - Vic specific module	20/09/2021	1.0
Other	F21-407 GTLR UOM RRC Approval	20/09/2021	1.0
Other	UniMelb Ethics Response 26th October 2021	26/10/2021	1.0
Consent form	2. Men in Mind trial - standard PICF_revised	26/10/2021	1.0

Conditions of Approval:

Research projects are normally approved to the anniversary date of the approval. Projects may be renewed yearly for up to a total of three years upon receipt of a satisfactory annual report. If a project is to continue beyond three years, two optional extensions of one year each (3+1+1) will need to be applied for. Anything beyond 5 years will need a new application to be submitted.

Please note that the following conditions apply to your approval. Failure to abide by these conditions may result in suspension or discontinuation of approval and/or disciplinary action.

1. **Limit of Approval:** Approval is limited strictly to the research as submitted in your Project application.
2. **Variation to Project:** Any subsequent variations to the Project must be notified formally to the Committee for consideration and approval before they are implemented. If the Committee considers that the proposed changes are significant, you may be required to submit a new application.
3. **Incidents or adverse events:** Researchers must report immediately to the Committee anything that could affect the ethical acceptability of the project, including adverse effects on participants or unforeseen events. Failure to do so may result in suspension or cancellation of approval.
4. **Monitoring:** All projects are subject to monitoring at any time by the Committee.
5. **Annual Report:** An annual report must be submitted each year on the anniversary of project approval, and at the conclusion of the project. Ethics approval will lapse if an annual report is not submitted.
6. **Auditing:** All projects are subject to audit by members of the Committee.
