# Supplementary material for: Protocol for a randomized controlled trial of the Men in Mind training for mental health practitioners to enhance their clinical competencies for working with male clients
Source: BMC Psychol. 2022 Jul 15;10:174. doi: 10.1186/s40359-022-00875-9 (PMC9288021; doi:10.1186/s40359-022-00875-9)
Supplement: Supplementary file 3 — Additional file 3: Funding statement. [file 40359_2022_875_MOESM3_ESM.pdf]

Australian Government

Department of Health

National Health and
Medical Research Council

In reply please quote: NHMRC 2019 MRFF Million Minds Mission Suicide Prevention 2020 Application APP1199972

Professor Jane Pirkis
j.pirkis@unimelb.edu.au

Dear Professor Pirkis

Application ID: APP1199972
Type: 2019 MRFF Million Minds Mission Suicide Prevention 2020
Application Title: Preventing suicide in boys and men

I am pleased to advise that your application (APP1199972) for MRFF - Research Grants 2019 MRFF Million Minds Mission Suicide Prevention 2020 funding commencing in 2020 has been approved.

This letter provides you with important information about the Offer of Funding being made to University of Melbourne for this application.

The offer

The Offer of Funding for your Application is made subject to the execution of a Funding Agreement for funding of grants from the MRFF Health Special Account (the Funding Agreement). This Funding Agreement may be in place, or will shortly be provided to your Administering Institution for signature. Your Research Administration Office (RAO) will be required to execute a Schedule in RGMS that specifies the approved budget and the due date for acceptance of the offer.

The Funding Agreement will be between the Australian Government and your Administering Institution. Your Administering Institution is responsible for informing you about the requirements of the Funding Agreement (including its Schedules), the Direct Research Costs guidelines and other applicable policies.

Assessment Details

Further information about the assessment of your application is provided separately to the RAO at your Administering Institution and they can supply this to you.

All enquiries relating to your application should be directed to the NHMRC through the RAO at your Administering Institution.

Yours sincerely

[Authorised for electronic transmission]

Alan Singh
Executive Director
Research Translation Branch
